# Exploring the predictive properties of the Hayes Ability Screening Index subtest background information in identifying individuals with MBID among in-patients with SUD

**DOI:** 10.3389/fpsyt.2022.1051946

**Published:** 2022-12-15

**Authors:** Kirsten J. Braatveit, Jörg Assmus, Oddbjørn Hove

**Affiliations:** ^1^Department of Research and Innovation, Helse Fonna HF, Haugesund, Norway; ^2^Blue Cross Clinic Haugaland, Addiction Treatment Centre, Haugesund, Norway; ^3^Helse Bergen HF, Haukeland University Hospital, Bergen, Norway

**Keywords:** intellectual disability, borderline intellectual disability, learning difficulties, substance use disorder, inpatient, screening

## Abstract

**Introduction:**

For individuals with substance use disorder (SUD), mild to borderline intellectual disability (MBID) goes undetected in treatment clinics. The Hayes Ability Screening Index (HASI) has been found to be a valid, time-saving screening instrument for MBID in SUD treatment. MBID can have significant implications for treatment planning and outcomes. Therefore, it is important to have methods for the early recognition of these comorbid conditions. Because of less sensitivity to recent or ongoing substance use, the HASI subtest background information may be particularly valuable as an early screening of MBID. The main aim was to investigate the convergent, predictive, and discriminant validity of the HASI subtest background information in identifying in-patients with SUD as MBID or non-MBID.

**Methods:**

Eighty-four in-patients with SUD aged 19–64 participated in this multicentre study. MBID was diagnosed according to the ICD-10 using WAIS-IV, Vineland II, and self-reported childhood learning difficulties.

**Results:**

The main finding was that, among the HASI subtests, background information was the strongest predictor. A HASI background information cut between 6 and 7 showed a sensitivity of 78% and a specificity of 72%.

**Conclusion:**

The HASI subtest background information has acceptable convergent, predictive, and discriminant validity as a screening for MBID among in-patients in SUD treatment.

## Introduction

The identification of primary illness and comorbid conditions is important for the clinical understanding of patients’ needs in treatment planning for substance use disorder (SUD). One condition that can have major implications for the planning and management of SUD treatment is intellectual disability. There has been growing recognition of SUD in individuals with mild to borderline intellectual disability (MBID) (1). Studies have identified an overrepresentation of MBID in institutions offering treatment for SUD, with prevalence rates of up to 39% among in-patients (2, 3). Although these patients’ intellectual disabilities have been identified in SUD research, it is common that the disabilities are unrecognized at all levels of the clinical pathway, including both primary and secondary SUD as well as mental health services (2, 4–8). One reason for this may be the lack of tools for identifying impaired functioning associated with intellectual disabilities, regardless of the patient’s substance use at the time of referral or in the initial phase of the specialized SUD treatment, whether as in-patients or out-patients. Studies in the field have reported that individuals with MBID have negative experiences with mainstream SUD treatment (9), higher dropout rates from mainstream SUD treatment (10, 11), experience barriers to SUD treatment (10–12), elevated psychological stress (13), and higher rates of relapse to substance use during treatment (2). These findings suggest the need for the early recognition of MBID in SUD treatment because this may have a significant impact on the SUD treatment outcome.

To meet the criteria for intellectual disability, both intellectual functioning and adaptive behavior, as measured by standardized tests, must be impaired, and the level of functioning must have been present during the developmental period (22 years) (14). However, the use of an intelligence test is not straightforward in the SUD group. These patients are often under the direct or indirect influence of substances at the time of admission, which has unpredictable effects on the test results, consequently lowering the reliability and validity of the IQ measure. For patients with SUD, there are no guidelines regarding the timing of IQ testing. Ideally, intellectual assessment in individuals with SUD should be done when at least the short-term effects of substance use have a minimum influence on the results (e.g., cannabinoid 1 receptors regain normal functioning within 4 weeks and are associated with a reduction of withdrawal symptoms that can influence cognitive performance) (15). Significant cognitive improvement has been found within 2–6 weeks of abstinence from substance use (16), and for the specific measure of IQ, there seems to be no influence of substance use after 6 weeks (17). On the other hand, waiting too long before starting the assessment can increase the risk that patients with MBID will not be available for testing because of dropout in treatment. Cognitive deficits are one of the most consistent findings of risk factors for dropout from SUD treatment (18). A time gap of at least 2–6 weeks after admission before addressing possible MBID may have negative implications on patient satisfaction, the prevention of dropout, and the outcome of the treatment.

The Hayes Ability Screening Index (HASI) has been shown to be a valid and time-saving screening instrument for intellectual disability (ID) in the SUD population (19, 20) and has been shown to identify people with borderline intellectual disability (BID) as well (19). The HASI consists of four subtests measuring previous indications of learning difficulties (background information), backwards spelling, visuospatial (puzzle), and visuoconstructional (clock drawing) abilities (21). It has been found to correlate well with standardized measures of intellectual functioning and adaptive behavior (7, 8, 19, 20, 22). Previous studies have taken the possible influence of recent substance use into account and found the index to be a valid measure at 2–6 weeks of abstinence (19, 20). As for an even earlier identification that would be more relevant for referral/treatment planning and for a fuller understanding of patients who struggle to achieve or retain abstinence, there might be the same problem with the HASI as for the measures of intelligence above; most of the subtests on the HASI rely on the domains that may be influenced by ongoing or recent substance use, such as motor, sensory, or cognitive functioning. However, the subtest background information has potential because it is not a performance task, but rather, it is historical; thus, this test is not sensitive to ongoing substance use in similar ways to the other three. It consists of four questions related to learning difficulties (e.g., “Do you think that you are a slow learner?” “Have you received special education or been in a class for students that need extra help because of learning difficulties?”). As opposed to the other three, information on this subtest may also be collected from third parties or medical records, which do not rely on the patients’ “here-and-now” responses. Background information can be collected at any point in the clinical pathway, thus paving the way for a cognitively sensitive referral and intake process in specialist services.

Little is known about the subtest background information’s predictive abilities for patients with SUD or compared with the other subtests of the HASI. A study from a SUD population reported a statistically significant correlation between background information and measures of intelligence (20). To et al. (20) also found statistically significant correlations for the other HASI subtests. However, they did not investigate the subtest’s predictive or discriminant validity. As predictive validity provides useful data about test validity because it has greater fidelity to the real situation in which the test will be used than other validity tests, these analyses may be particularly useful in the context of the early detection of possible MBID in patients with SUD. Screening for MBID using questions about learning difficulties, special education, and disability pension, as in the background test, has high ecological validity.

The main aim of the current study was to investigate the convergent, predictive, and discriminant validity of the HASI subtest background information in identifying in-patients with SUD as MBID or non-MBID. The study also included the other HASI subtests in the analysis because this has not previously been studied.

## Materials and methods

### Study design

The study applied a retrospective, cross-sectional design. The timing of measurements on the Wechsler Adult Intelligence scale – fourth edition (WAIS-IV), the HASI, and the Vineland Adaptive Behavior Scale – second edition (Vineland II) was part of the design. We conducted the WAIS-IV and HASI assessments after 6 weeks of abstinence to minimize the influence of substance use. The Vineland II was administered at the end of treatment for scoring based on the most possible behavioral observations, resulting in results that reflect mean functioning rather than single episodes of functioning.

### Participants

The study population consisted of individuals over the age of 18 who were admitted to mainstream in-patient SUD treatment. All participants were diagnosed with a SUD for one or more substance(s). Several participants had been diagnosed with comorbid mental illness. A further description of participant characteristics is included in the results section. The exclusion criteria were having another first language than Norwegian, having been tested with the Wechsler Adult Intelligence Scale (WAIS) within the past 6 months prior to inclusion in the study, and being under the influence of substances on the day of testing.

Three out of five invited treatment facilities for SUD in Norway participated in the recruitment of patients and provision of patient information. The three facilities participated based on willingness. All three facilities were part of the public health system in Norway and followed the recommended national guidelines for SUD treatment (23, 24). Neither invited facilities nor patients received any compensation for their participation.

### Measures

The Norwegian translation of the WAIS-IV was used to determine the participants’ IQ. The study relied on Scandinavian norms. The test has been shown to have good reliability and validity (25). In the current study, results on the level of full-scale IQ (FSIQ) were used.

The Norwegian translation of the Vineland II was used as a measure of the participants’ adaptive behavior. The measure can be interpreted at the level of the global adaptive score (GAS), as well as the more specific domains of communication (CF), daily living (ADL), and socialization skills (SF). The reliability and validity of the scale were studied thoroughly and have been found to hold good psychometric properties (26).

The Norwegian version of the HASI was administered as a screening for ID among the participants. It consists of four subtests: background information (BI), with four questions related to learning difficulties, each scored as 1 or 2 (sum; 4–8), backwards spelling (BS), with a minimum raw score of 1 and maximum of 5, puzzle (PZ), with a minimum raw score of 1 and maximum of 2, and clock drawing (CD), with a minimum raw score of 1 and a maximum of 10. Raw scores are converted to scaled scores, and a sum of 26 is added to the scaled score for the patients’ total score on the HASI. The minimum total score of the HASI is 38.2, and the maximum score is 99. For adults, a cut-off score of 85 is set, and the results under this cut-off indicate the need for further ID assessment. The instrument has been found to have good psychometric properties, both in the original version (21) and in the Norwegian version (27). The instrument has been validated for ID screening in the SUD in-patient population using the current sample of participants (19).

A self-report questionnaire was used to obtain information on historical and demographic variables concerning, among others, current and previous substance use, childhood learning difficulties (range 0–7), education (1 = completed primary school, 2 = completed secondary school, 3 = completed further education, and 4 = completed higher education), and a sum score of contact with public health and support systems during childhood/adolescence (range 0–5), including pedagogical services (yes/no), child psychiatric services (yes/no), youth AUD/SUD services (yes/no), child protection services (yes/no), and police (yes/no). Many of the questions, including those regarding current and previous substance use, were derived from a national questionnaire for all patients receiving SUD treatment in Norway (28).

A severity of substance use index was calculated by summarizing the items on regular substance use before the age of 16 (yes/no), syringe use in the last 4 weeks prior to treatment admission (yes/no), lifetime overdose (yes/no), prior SUD/AUD treatment (yes/no), and polydrug use (yes/no) (two or more substances used regularly in the last 4 weeks prior to admission) into a variable ranging from 0 to 5, where 5 indicates the most severe use. The number of drug relapses during the treatment period was recorded by the treating psychologist/physician.

The seven questions included as childhood learning difficulties (e.g., “I was slow in learning to talk” and “I had immature behavior for my age”) were a result of a confirmatory factor analysis of 40 items that suggested adequate model fit for a five-factor model, grouping different childhood difficulties as learning difficulties and four other categories (17). To indicate learning difficulties during childhood in classifying MBID, the participants had to reply “yes” to at least one of the questions on previous learning difficulties.

A therapist questionnaire was used to collect the data on the participants’ diagnosis, relapse to substance use during treatment, the reason for treatment termination, length of abstinence at treatment termination, and further public or private support after treatment. Standardized clinical procedures that included structured interviews, such as the Mini-International Neuropsychiatric Interview (M.I.N.I) and the Structured Clinical Interview for DSM disorders (SCIDs), were used to assess both mental illness and substance use disorders. A diagnosis was made when the participant met the ICD-10 criteria (29).

### Classification of ID

To be labeled as having an ID, the participants had to meet the criteria for *definite*, *probable*, or *possible* ID. To qualify for a *definite* ID, the participants had to meet all three diagnostic criteria for an ID. The full-scale IQ (FSIQ) had to be 69 or lower; similarly, the GAS on the Vineland II or at least one of the specific domains had to be 69 or lower, and there had to be an indication of learning difficulties before the age of 18. To qualify for a *probable* ID, there had to be indications of childhood learning difficulties, GAS, or at least one specific domain on the Vineland II measured at 69 or lower, along with an FSIQ on the WAIS-IV of 70–73, accounting for the standard error of measurement. A *possible* ID was identified when information on only one or two of the diagnostic criteria for ID was available, and the results indicated ID.

The same procedure was used to label the BID. To qualify for a *definite* BID, the FSIQ had to be between 70 and 85, the GAS or at least one of the specific domains on the Vineland II had to be in the range of 70–85, with none being 69 or lower, and there had to be an indication of learning difficulties before the age of 18. For a *probable* BID, information on all three criteria for BID was available, but either the FSIQ or Vineland II results had to indicate ID. To qualify for a possible BID, only one or two of the diagnostic criteria were available and indicated functioning on the BID level. For more information about ID and BID case profiles, refer to Braatveit et al. (2).

### Procedures

Upon admission to treatment, all patients were invited to participate in the study. The participants signed an informed consent form and received the self-report questionnaire to answer. Missing data on the self-report questionnaire in eight cases were supplemented by journal data.

A WAIS-IV and HASI assessment appointment was scheduled 6 weeks after the last intake of substances. A clinical psychologist or student of psychology administered the instruments. Patients’ abstinence from substance use was ensured through urine samples and clinical observations. At the end of the participants’ treatment program, the treating psychologist/physician answered the therapist questionnaire, and personnel at the institution completed the Vineland II based on observations done throughout the entire treatment period. They were encouraged to complete the scale in groups to base the scoring on a wide range of observations.

### Staff training and interrater reliability

For all participants, a clinical psychologist or student of psychology employed at the participating institution administered the WAIS-IV and the HASI. The administrators received training through a 3-day theoretical and practical course on the administration and scoring of the two instruments. A clinical psychologist competent in the administration, scoring, and interpretation of the instruments gave the course. After the course, the administrators scored the same nine videos of patients’ responses on four randomly selected WAIS-IV subtests and the HASI. Intraclass correlation (ICC) analysis was calculated and showed results between 0.84 and 1.0 for the WAIS-IV and 0.75 and 0.98 for the HASI. The ICC results indicated almost perfect consistency among raters on the WAIS-IV and substantial to almost perfect on the HASI, according to Landis and Koch (30).

### Statistical methods

The SPSS 26 (IBM Corp., Armonk, NY) statistical software package was used for all statistical analyses. The interitem correlations, the convergent validity of the HASI subtests, and WAIS-IV the HASI subtests and Vineland II were assessed using a Pearson two-tailed correlation test. The predictive properties of the HASI subtests for MBID were assessed using binary logistic regression analysis. The discriminative ability of the HASI subtests was investigated using a receiver operating characteristics (ROC) curve analysis.

### Ethics

The Regional Ethical Committee for Medical Research in Norway approved the study (Reference: 2011-00778).

## Results

### Recruitment and participant flow

**FIGURE 1:**
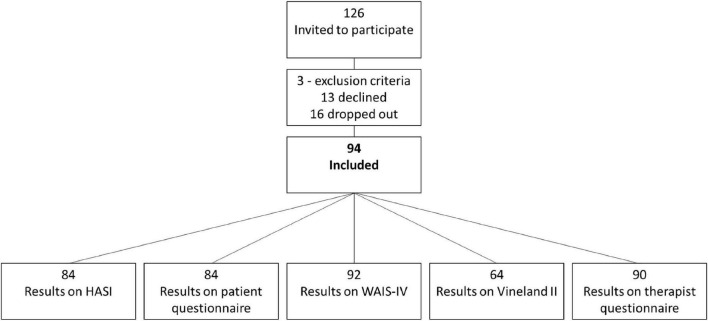
Overview of the recruitment process.

During the recruitment period of 2013–2015, a total of 126 patients were invited to participate in the study. Thirty-one participants had complete cases regarding all three ID measures. Because we operated with different levels of certainty regarding ID/BID (refer to the methods section) and participants could have one or two of the three criteria missing, a sample of 91 was eligible for classification as ID/BID or non-ID/BID. Eighty-four participants had completed the HASI. Thus, the final sample eligible for the binary logistic regression analysis and ROC curve analysis was 84. Refer to Figure 1 for a simple overview.

### Description of the sample

The sample characteristics are shown in Table 1. Of the 84 participants included in the analysis, 33.3% were female participants, their mean age was 33.31 (*SD* = 11.6), and their mean age of the first episode of substance use was 13.8 (SD = 1.8).

**TABLE 1:** Description of the sample.

Characteristics	Valid *N*	Value
Age^1^	84	33.3 (11.6)
Age first episode use^1^	84	13.8 (1.8)
Gender (Female)^2^		28 (33.3%)
Education^2^^,^^3^	78	
Primary school (1st-6th grade)		5 (6.4%)
Secondary school (7th-9th grade)		42 (53.8%)
Further education (10th-12th grade)		31 (39.8%)
Higher education		0
Number of mental disorder^1^	93	0.6 (0.5)
Number of SUD diagnosis^1^	93	2.2 (1.2)
Disorder	93	
Any mental disorder (F20-F90)	58 (62.4%)	
One SUD		34 (36.6%)
Two or more SUD		59(63.4%)

^1^Mean (SD).

^2^N (%).

^3^Highest completed education.

The comparison of the MBID and non-MBID groups is shown in Table 2. The differences between the MBID and non-MBID for all considered variables (HASI, WAIS-IV, and Vineland II) were significant (all *p* = 0.001 or lower).

**TABLE 2:** Mean values on the HASI, WAIS-IV, and Vineland II for the MBID and no-MBID group.

	MBID			Normal			Test statistics		
	*N*	M^4^ (SD)^5^	Min-max	*N*	M^4^ (SD)^5^	Min-max	*t*-value	df	*P*-value^1^
HASI total^3^	27	77.2 (9.6)	53-92	57	89.6 (5.3)	74-96	7.60	82	<0.001
Subtests^1^^,^^2^	27			57					
HASI background		5.7 (1.1)	4-8		7.11 (1.1)	4-8	5.50	82	<0.001
HASI backwards spelling		4.1 (1.6)	0-5		4.9 (0.5)	3-5	3.48	82	0.001
HASI puzzle							4.46	82	<0.001
HASI clock-drawing		7.7 (2.3)	3-10		9.0 (1.4)	4-10	3.35	82	0.001
WAIS-IV FSIQ^3^	26	74 (6.7)	61-84	57	92 (10.3)	72-118	8.86	81	<0.001
Vineland II global^3^	18	77 (10.4)	50-92	39	104 (12.4)	66-120	6.81	55	<0.001
Communication^3^	18	73 (12.9)	48-104	40	102 (14.2)	65-114	7.62	56	<0.001
Daily living^3^	18	86 (12.5)	64-112	40	105 (12.9)	57-123	5.35	56	<0.001
Socialization^3^	18	80 (14.1)	55-107	40	101 (10.6)	73-115	6.34	56	<0.001

^1^Chi-square for HASI puzzle (X^2^(1, 84) = 18.71, *p* ≤ 0.001), otherwise *t*-test.

^2^Raw scores.

^3^Index score.

^4^Mean.

^5^Standard deviation.

Based on the classification procedures for classifying ID and BID using all three diagnostic criteria described in the methods section, *n* = 6 (7.1%) were classified as ID, *n* = 21 (25%) as BID, and *n* = 57 (67.9%) as non-MBID.

### Interitem correlations

The relationship between the HASI subtests was calculated using the Pearson two-tailed correlation test for the raw scores. The results showed a correlation between all the subtests with coefficients ranging from 0.22 to 0.35. The results are shown in Supplementary Table 1.

### Convergent validity

Here, convergent validity was calculated using the Pearson two-tailed test for the correlation between HASI scores and the WAIS-IV and the Vineland II. The results showed a correlation between all of the HASI subtests and the full-scale WAIS-IV, ranging from 0.35 to 0.54. For the Vineland II, the HASI BI subtest correlated with the most indexes, with coefficients ranging from 0.49 to 0.32. For an overview of the results of the convergent validity, refers to the lower part of Supplementary Table 1.

### Predictive validity

#### Group prediction

As shown in Table 3, the subtests BI (*p* = 0.004) and puzzle (*p* = 0.022) significantly predicted the classification as MBID vs. non-MBID in the multivariate model. Here, the standardized odds ratio (OR) of the BI [2.6, 95%CI = (1.4–5.1)] was somewhat higher than for the puzzle subtest [2.3, 95%CI = (1.1–4.6)]. However, the univariate models showed that all subtests alone had a significant association with the classification (all *p*-values of 0.01 or less).

**TABLE 3:** Binary logistic regression (*N* = 84).

	Unstandardized variables	Standardized variables	
Univariate model	OR^1^ [C.I (95%)]	OR^1^ [C.I (95%)]	*P*-value
Background	2.9 (1.8–4.8)	3.7 (2.0–6.9)	< 0.001
BW spelling	2.3 (1.2–4.4)	2.4 (1.2–4.7)	0.010
Puzzle	11.0 (3.0–40.8)	3.3 (1.7–6.3)	< 0.001
Clock drawing	1.5 (1.1–2.0)	2.1 (1.3–3.5)	0.004

**Full model**	**OR [C.I (95%)]**	**OR [C.I (95%)]**	***P*** **-value**

Background	2.2 (1.3–3.8)	2.6 (1.4–5.1)	0.004
BW spelling	1.7 (0.7–4.0)	1.7 (0.7–4.2)	0.257
Puzzle	5.2 (1.3–21.3)	2.3 (1.1–4.6)	0.022
Clock drawing	1.4 (1.0–2.1)	1.9 (0.9–3.8)	0.083

^1^Odds ratio.

#### Discriminative validity

Based on the raw scores, the ability of the HASI background subtest to discriminate between those with and without MBID was calculated using ROC curve analysis. The area under the curve was 0.807 (95% *CI* = 0.703–0.912), thus indicating an acceptable to excellent ability to discriminate between the two groups. Using a Euclidean distance, a cut between 6 and 7 showed a sensitivity (0.778) and specificity (0.702) closest to the ideal point (sensitivity 1 and specificity 1), suggesting the cut-off.

An additional ROC analysis was run for the instruments’ total scores’ ability to discriminate the MBID group from the non-MBID group. The area under the curve was 0.886 (95% *CI* = 0.808–0.963), indicating an excellent ability to discriminate between the two groups. The sensitivity was 0.815, and the specificity was 0.825, at the original HASI cut-off score of 85.

#### Factors associated with MBID

For a comparison with the same factors associated with MBID when using the full diagnostic criteria (2), several analyses were run for a HASI background cut-off score of 7 (refer to discriminant validity). For the results, refer to Table 4.

**TABLE 4:** Factors associated with MBID.

	MBID		Normal		
Factors	*n*	Value (Value)	*n*	Value (Value)	*P*-value
Public support system^1^	35	2.17 (1.65)	32	1.00 (1.41)	0.003*
Age first use^1^	35	13.69 (1.78)	34	13.90 (1.80)	0.699
Age regular use^1^	32	16.25 (2.95)	34	16.86 (4.94)	0.543
Years from first to regular use^1^	31	2.77 (2.36)	32	3.42 (4.63)	0.486
Number for SUD^1^	31	2.16 (0.97)	52	2.09 (1.22)	0.801
Severity of use^1^	36	2.33 (1.37)	37	2.43 (1.19)	0.743
Number of mental disorders^1^	37	2.14 (0.98)	46	2.11 (1.25)	0.916
Education^2^	24	2 [1-5]	39	2 [1-5]	0.102
Childhood learning difficulties^2^	24	1 [0-4]	39	0 [0-5]	0.006
Mental disorders^3^					
Psychosis disorders (F2x)	31	2 (6.5)	52	1 (1.9)	0.282
Affective disorders (F3x)	31	4 (12.9)	52	11 (21.2)	0.345
Anxiety disorders (F4x)	31	7 (22.6)	52	9 (17.3)	0.556
Personality disorders (F6x)	31	2 (6.5)	52	4 (7.7)	0.883
Developmental disorders (F8x)	31	6 (19.4)	52	2 (3.8)	0.021*
Conduct disorders (F9x)	31	9 (29.0)	52	5 (9.6)	0.022*
Relapse during treatment^3^	30	13 (43.3)	51	11 (21.5)	0.038*
Types of drugs^3^					
Alcohol	31	8 (25.8)	52	24 (46.2)	0.065
Opiates	31	12 (38.7)	52	13 (25.0)	0.188
Cannabis	31	12 (38.7)	52	22 (42.3)	0.747
Sedatives	31	14 (45.2)	52	21 (40.4)	0.670
Cocaine	31	1 (3.2)	52	1 (1.9)	0.708
Stimulants	31	19 (61.3)	52	23 (44.2)	0.133
Hallucinogenic	31	0 (0.0)	52	1 (1.9)	0.437
Multiple drugs	31	1 (3.2)	52	4 (7.7)	0.408

^1^*Mean (SD), *t*-test.

^2^Median [Min-max], Manfred Whitney U test.

^3^*n* (%), Chi-square test.

## Discussion

The literature has indicated good validity of the HASI total score for identifying ID in the SUD population at the original cut-off (19, 20) and that a large number of those falsely identified as ID by the HASI had an IQ under 85, thus falling into the category of borderline intellectual functioning (19). The current study investigated the convergent, predictive, and discriminant properties of the HASI subtests, and the main finding was that the HASI subtest BI was the strongest predictor in the classification of MBID. The HASI subtest BI has a fair predictive strength for MBID classification. This subtest showed one of the strongest correlations with the WAIS-IV and the most correlations with the Vineland II. The effect size of the relationship between HASI BI and the classification of MBID was small to moderate. Measures like a review of medical records for the indicators of developmental or learning difficulties during childhood and comparative information from sources that know the patient well, for example, from the developmental period or school history, can add to the reliability of the background test. Our present findings suggest that the BI subtest was slightly less sensitive and specific than the full HASI for the MBID group. Considering the low effort needed to administrate the BI, it is our overall conclusion that the HASI subtest BI has acceptable validity for screening MBID among in-patients with SUD when the administration of the full-scale HASI is not applicable or most likely would result in an invalid result. Our data suggest using a cut-off score < 7 for the BI subtest, where scores of 6 or lower indicate the need for further assessment. To have an early indication of MBID can be of clinical value for patients with MBID because they are known to have higher treatment dropout rates and more negative treatment experiences than the general SUD in-patient group.

Our correlation findings align with a previous study on a SUD population, in which all of the HASI subtests correlated statistically significantly with the measure of intelligence (20). Our findings deviate from a previous study on a psychiatric population in which a non-significant correlation was found between the HASI subtest background and WASI measures of intelligence (7). The authors suggest that individuals underreport signs of MBID because the condition is associated with stigma. For the SUD population, underreporting does not seem to be the case because the current study found correlations between the HASI background and most of the WAIS and Vineland indexes. It is not clear in the article of Søndenaa et al. (7) as to the recruitment process, where the patient’s conditions or diagnoses were unknown. Thus, we cannot rule out that selection bias has influenced their findings. In addition, they used a measure of intelligence as a validity criterion that has uncertain validity for the Norwegian population (31). Therefore, future studies should aim at clarifying the correlation between HASI BI and intelligence-validated intelligence measures for the psychiatric population.

Correlating the HASI background using a cut-off score of <7 with factors previously found to be associated with MBID when using the full diagnostic criteria [refer to (2)], we found similar associations with most factors. This included a higher rate of childhood learning difficulties, more contact with public support systems during the developmental period, and more relapse to substance use during treatment for the MBID group than for the non-MBID group. Some differences in the findings were found regarding education and the type of mental disorder. These differences might be because of a small sample and a less sensitive and specific classification method than using the diagnostic criteria for group membership as MBID or non-MBID. However, the vast majority of the factors associated with MBID when using the HASI background cut-off score of <7 was the same as when using the full diagnostic criteria, indicating a fair predictive ability of the associated factors and in the prediction of group membership (MBID vs. non-MBID).

## Strengths and limitations

The main strength of the current study was the use of all three ICD-10/DSM-5 diagnostic criteria for MBID classification as validation criteria for the HASI subtests. Previous studies on MBID and SUD have mainly used the criteria of intellectual functioning, leaving the classification of MBID with shortcomings. The current study also included a large clinical sample of in-patients in SUD treatment. Few previous studies on MBID and SUD have studied patients in SUD treatment with a fully developed substance use disorder. Also, the current study ensured a minimum period of 6 weeks of abstinence to minimize the potential influence of ongoing or recent substance use on test results.

The results of the present study should also be interpreted in the context of some limitations. Although the current study found the BI subtest to predict MBID at 6 weeks of abstinence, it is not known how reliable subjects’ responses are during ongoing or recent substance use. The current study cannot conclude with its predictive abilities in the early weeks of SUD in-patient treatment or for SUD outpatients. Future studies should investigate this further. Although the classification procedures of the current study were strict, only 31 participants had complete results on all three measures. Cases with missing information on one or two of the criteria have left the classification results with some uncertainty, so they should be interpreted with caution. In addition, for the criteria of onset of the developmental disorder, the present study used a scale for childhood difficulties that had not been independently validated, although a confirmatory factor analysis suggested an adequate model fit (17).

## Conclusion

The HASI subtest background has acceptable convergent, predictive, and discriminant validity as a screening for MBID among in-patients in SUD treatment. MBID is prevalent in SUD treatment but often goes undetected, thus, all patients should be screened for the condition. We encourage clinicians to collect information on the four questions when it is not possible to administer the full HASI. We further recommend that patients be considered for a full MBID assessment if their raw score on the HASI subtest BI is 6 points or lower.

## Data availability statement

The raw data supporting the conclusions of this article will be made available by the authors, without undue reservation.

## Ethics statement

The studies involving human participants were reviewed and approved by Regional Etisk Komite Norge. The patients/participants provided their written informed consent to participate in this study.

## Author contributions

KB had the mail work with the whole manuscript, including writing all the sections. OH had contributed significantly in commenting and re-writing of the manuscript, suggesting angles, methods for analysis and interpretation of results. JA had mainly contributed on statistical methods and interpretation of results. All authors contributed to the article and approved the submitted version.
