# Supplementary material for: Exploring the predictive properties of the Hayes Ability Screening Index subtest background information in identifying individuals with MBID among in-patients with SUD
Source: Front Psychiatry. 2022 Dec 15;13:1051946. doi: 10.3389/fpsyt.2022.1051946 (PMC9797666; doi:10.3389/fpsyt.2022.1051946)
Supplement: Supplementary file 1 [file Table_1.docx]

|  |  |  |  |  |  |  |
| --- | --- | --- | --- | --- | --- | --- |
| Table S1. Correlations between the HASI subtest, WAIS-IV and Vineland II | | | | | |  |
|  |  |  |  |  |  |  |
|  |  | HASI | | | | |
|  |  | Background | BW spelling | Puzzle | Clock drawing | Total Score |
| HASI | |  |  |  |  |  |
|  | Background | 1 | .34 (.13-.51) | .35 (.15-.53) | .23 (.01-.42) | .73 (.61-.82) |
|  | BW spelling | - | 1 | .22 (.01-.42) | .37 (.17-.54) | .68 (.55-.78) |
|  | Puzzle | - | - | 1 | .22 (.00-.41) | .55 (.37-.68) |
|  | Clock drawing | - | - | - | 1 | .67 (.53-.77) |
|  |  |  |  |  |  |  |
| WAIS FSIQ | | .53 (.36-.67) | .35 (.15-.53) | .54 (.37-.68) | .46 (.27-.62) | .70 (.56-.80) |
| Vineland GAS | | .49 (.28-.66) | .20 (-.06-.43) | .23 (-.04-.46) | .33 (.06-.54) | .50 (.27-.67) |
| Vineland CF | | .42 (.18-.61) | .23 (-.03-.46) | .15 (-.10-.40) | .41 (.17-.61) | .51 (.28-.68) |
| Vineland ADL | | .21 (-.05-.45) | .03 (-.23-.28) | .27 (.01-.49) | .19 (-.07-.43) | .26 (.01-.49) |
| Vineland SF | | .32 (.07-.53) | .15 (-.11-.40) | .31 (.05-.52) | .18 (-.08-.42) | .36 (.12-.57) |
